# Risk factors for the transmission of *Clostridioides difficile* or methicillin-resistant *Staphylococcus aureus* in acute care

**DOI:** 10.1017/ash.2023.376

**Published:** 2023-09-29

**Authors:** Xuetao Wang, Matthew Garrod, Tamara Duncombe, Eunsun Lee, Katy Short Joyce Ng

## Abstract

**Background:** Some hospitals continue to struggle with nosocomial transmission of *Clostridioides difficile* infection (CDI) and methicillin-resistant *Staphylococcus aureus* (MRSA) despite years of infection control efforts. We investigated the relationship between unit infrastructural–organizational risk factors and nosocomial transmission of CDI and MRSA. **Methods:** This retrospective observational study included 100 eligible acute-care inpatient units from 12 hospitals in British Columbia, Canada, from April 1, 2020, to September 16, 2021. The outcome variables included whether a unit was on the CDI or MRSA vulnerable unit list (ie, defined as having ≥5 CDI cases or ≥6 MRSA cases being attributed to the unit in the last 6 fiscal periods), the average CDI/MRSA rate, as well as the average CDI/MRSA standardized infection ratio (SIR). Independent variables included, but were not limited to, infection control factors (eg hand hygiene rate), infrastructural factors (eg, unit age, total beds on unit), and organizational factors (eg, hallway bed utilization, nursing overtime). Multivariable regression was performed to identify statistically significant risk factors using SAS, R Studio, and Stata software. **Results:** For CDI, older units were associated with higher odds of being on the CDI vulnerable unit list (aOR, 1.086; 95% CI, 1.024–1.175), higher CDI rate (adjusted relative risk [aRR], 0.012; 95% CI, 0.004–0.020), and higher CDI SIR (aRR, 0.011; 95% CI, 0.003–0.020). Larger unit size was associated with higher odds of being on the CDI vulnerable unit list (aOR, 1.210; 95% CI, 1.095–1.400) and higher CDI SIR (aRR, 0.013; 95% CI, 0.001–0.026). For MRSA, an increase in hand hygiene rate was associated with lower odds of being on the MRSA vulnerable unit list (aOR, 0.71; 95% CI, 0.53–0.897), lower MRSA rate (aRR, −0.035; 95% CI, −0.063 to −0.008), and lower MRSA SIR (aRR, −0.039; 95% CI, −0.069 to −0.008). Higher MRSA bioburden was associated with higher odds of being on the MRSA vulnerable unit list (aOR, >999; 95% CI, >999 to >999), higher MRSA rate (aRR, 9.008; 95% CI, 5.586–12.429), and higher MRSA SIR (aRR, 4.964; 95% CI, 1.971–7.958). Additionally, higher MRSA rates were associated increased utilization of hallway beds (aRR, 0.680; 95% CI, 0.094–1.267), increased nursing overtime rate (aRR, 5.018; 95% CI, 1.210–8.826), and not having a clean supply room with the door consistently closed (aRR, −0.283; 95% CI, −0.536 to −0.03). **Conclusions:** Several infrastructural and organizational factors were associated with nosocomial transmissions of CDI and MRSA. Further research is needed to investigate the mechanisms by which these factors are associated.

**Disclosures:** None

